# Nutrient Sensing, Monitoring, and Control for Hydroponic Crop Production in Smart Greenhouses

**DOI:** 10.3390/s26206597

**Published:** 2026-10-17

**Authors:** Arnab Majumder, Md Nasim Reza, Sakib Robin, Ji-Yeon Kang, Sanghwa Lee, Hyeunseok Choi, Dong Hee Noh, Sun-Ok Chung

**Affiliations:** 1 Department of Agricultural Machinery Engineering, Graduate School, Chungnam National University, Daejeon 34134, Republic of Korea; majumderarnab@o.cnu.ac.kr (A.M.); reza5575@cnu.ac.kr (M.N.R.); 2 Department of Smart Agricultural Systems, Graduate School, Chungnam National University, Daejeon 34134, Republic of Korea; s.robin97@o.cnu.ac.kr; 3 Bonghwa Insect Hotel Agricultural Corporation, Ltd., Bonghwa 36218, Republic of Korea; insecthotel0941@gmail.com; 4 Chuni Farm, Buyeo 33202, Republic of Korea; mychriss0820@naver.com; 5 Korea Institute of Industrial Technology, Cheonan 31056, Republic of Korea; hchoi@kitech.re.kr; 6 Jeonbuk Regional Branch, Korea Electronics Technology Institute (KETI), Jeonju 54853, Republic of Korea; dhee.noh@keti.re.kr

**Keywords:** precision agriculture, smart greenhouse, hydroponics, nutrient sensing, ion-selective electrode, closed-loop control

## Abstract

Hydroponic crop production in smart greenhouses requires accurate nutrient monitoring and reliable control, especially in recirculating systems. This review critically examines sensing principles, data acquisition, control strategies, actuation, feedback, and system integration. Electrical conductivity (EC) and pH are widely used for dosing, but EC cannot quantify individual nutrient concentrations. Emerging technologies include ion-selective electrodes, solid contact and field effect sensors, optical spectroscopy, multi-sensor arrays, and Internet of Things platforms. Their deployment remains constrained by interference, drift, fouling, reference instability, calibration demands, and matrix effects. Control approaches range from threshold-based dosing and EC/pH feedback to proportional–integral–derivative, rule-based, ion-specific, and model-based strategies. Published ion-specific replenishment and hybrid EC–ion studies demonstrate feasibility under tested conditions. However, differences in nutrient formulations, study duration, and error definitions limit conclusions about commercial control accuracy. This review proposes an integrated framework linking sensing, decision making, actuation, and verification, with performance assessed at analytical, temporal, operational, and system/control levels. Future research should prioritize reliable multi-ion sensing, automated data quality checks, delivered dose verification, safe operation during faults, and communication between components. Validation should include complete crop cycles, maintenance, fault recovery, and independent sites. These requirements support evaluation of nutrient use efficiency, operational reliability, and scalability across crops and hydroponic systems under realistic production conditions.

## 1. Introduction

Hydroponic crop production has been increasingly incorporated into smart greenhouses because nutrient solution and root zone conditions can be monitored and adjusted independently of soil-related variability. However, the limited chemical buffering capacity of hydroponic systems makes crop performance particularly sensitive to deviations in nutrient concentration, pH, dissolved oxygen, temperature, flow conditions, and actuator performance [1,2,3,4]. The technological effectiveness of a smart greenhouse therefore depends on whether nutrient solution conditions can be measured accurately and translated into reliable control actions.

Nutrient solution composition changes continuously because plant uptake is selective, water loss alters solute concentrations, source water introduces background ions, and recirculation retains ions at rates that may differ from crop removal. EC may therefore remain within an acceptable range while individual nutrients decline or background ions accumulate. Spatial variation can also develop among the reservoir, supply line, root zone, and return flow because mixing and hydraulic conditions are not uniform [5,6,7]. These dynamics require repeated measurement and feedback-based adjustment rather than reliance on an initially prepared solution alone.

Conventional monitoring and control have relied on manual sampling and adjustment or automated control based primarily on EC and pH thresholds. Although these measurements are operationally useful, they do not provide a complete characterization of nutrient composition. EC represents the collective electrical conductivity of dissolved ions, whereas pH reflects hydrogen ion activity and the acid–base condition of the solution; neither measurement identifies which individual nutrient ions are deficient or excessive. Ion-specific concentrations can be determined through conventional laboratory analyses, but such approaches involve sampling, sample transport and preparation, analytical costs, and processing delays that limit their suitability for continuous control. Consequently, a technological gap remains between readily automated surrogate measurements and the ion-specific information required for precise nutrient adjustment [8,9].

Recent research has sought to address this gap through ion-selective electrodes (ISEs), spectroscopic sensors, multi-sensor platforms, automated sampling systems, wireless communication, programmable controllers, and feedback-based actuation. Related studies have examined EC/pH control, ion-specific replenishment algorithms, PLC-based automation, and IoT-enabled hydroponic systems [10,11,12,13]. However, the literature remains fragmented across measurement devices, data acquisition platforms, control algorithms, and actuation technologies. This fragmentation complicates evaluation of the complete pathway through which a sensor measurement is translated into a measurable and verifiable adjustment.

Recent reviews have examined complementary aspects of hydroponic nutrient monitoring and management. Ion-selective electrodes and IoT-based monitoring were synthesized by Richa et al. [14], whereas emerging ion-specific sensors were evaluated by Hong et al. [15]. Broader nutrient sensing trends were summarized by Reza et al. [9]; automated pH- and EC-dosing frameworks by Sulaiman et al. [13]; smart nutrient control models by Catota-Ocapana et al. [16]; and technological gaps in IoT-enabled platforms by Sportelli et al. [17]. Although these studies provide important insights into sensing, communication, or control technologies, comparatively limited attention has been given to the complete pathway through which measurements are validated, converted into control decisions, physically implemented through dosing and hydraulic equipment, and subsequently verified. The present review therefore examines the complete sensing–monitoring–decision–actuation–verification chain, including measurement reliability, calibration, controller strategies, dosing hardware, hydraulic mixing and transport delays, actuator verification, safety constraints, system interoperability, and readiness for continuous greenhouse operation.

A clear distinction between sensing, monitoring, control, and actuation is therefore required. In this review, sensing refers to the physical or chemical transduction of a nutrient-related variable into a measurable signal. Monitoring encompasses sensing together with sampling, signal acquisition, calibration, transmission, validation, visualization, and temporal observation. Control refers to comparison of the measured condition with a predefined target and generation of a corrective command. Actuation represents the physical implementation of that command through pumps, valves, injectors, circulation, mixing, or related equipment. Broader agronomic management was considered only where it directly defines requirements for a monitoring or control technology.

This review aimed (1) to critically examine and compare hydroponic nutrient monitoring technologies with respect to sensing principles, target variables, measurement performance, calibration requirements, operational limitations, and readiness for continuous greenhouse application and (2) to evaluate how measurements are translated into physical nutrient adjustments through control algorithms, dosing hardware, feedback mechanisms, and integrated monitoring–control architectures, thereby identifying the requirements for reliable closed-loop nutrient control in smart greenhouses.

## 2. Literature Search and Review Methodology

A structured critical review was conducted using PRISMA 2020 [18] as a reporting aid for identification and selection of the literature. The review compared nutrient monitoring technologies and examined how validated measurements are translated into nutrient adjustments through controller logic, dosing hardware, and feedback. The synthesis is qualitative; use of PRISMA terminology does not itself establish that independent duplicate screening, protocol registration, or a complete systematic review audit trail was performed.

**Figure 1:**
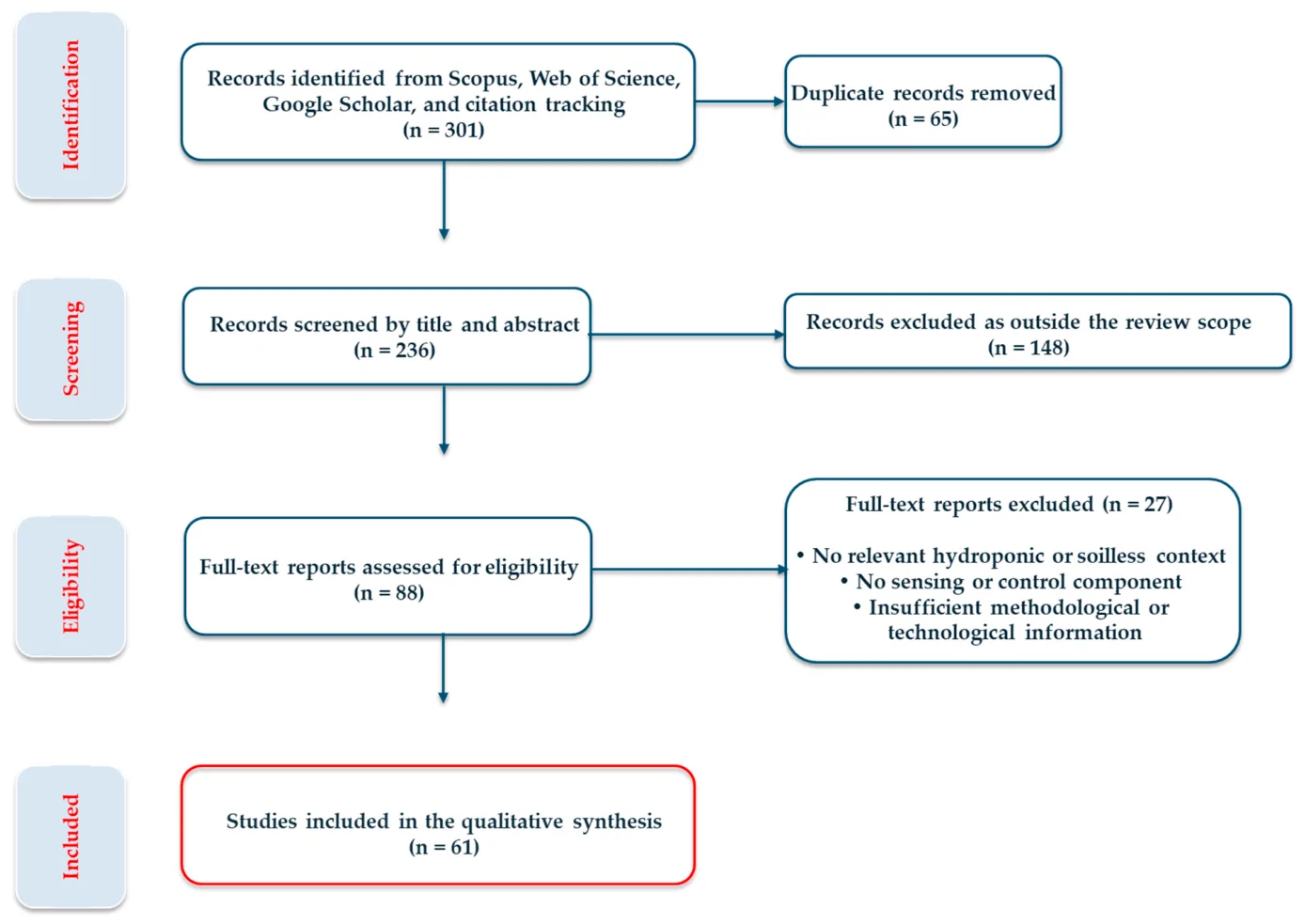
Literature selection flow reconstructed from the counts reported in Section 2.

Protocol registration and the date of the initial search are not documented in the available review record. These omissions limit reproducibility and prevent a claim of prospectively registered methods. PRISMA 2020 is used to organize reporting; Figure 1 remains a reconstruction of reported counts pending reconciliation with the original search and screening files.

Scopus and the Web of Science Core Collection were used as the primary bibliographic databases. Google Scholar was used as a supplementary source and for backward and forward citation tracking. The search covered the literature available up to 1 September 2026. The Scopus and Web of Science searches were rerun using the same strategy for the update period from 24 May to 1 September 2026; supplementary citation tracking identified one additional eligible study, which was incorporated into the review. No lower publication year limit was applied because both foundational publications and recent studies were needed to describe the development of nutrient sensing, monitoring, and control in hydroponic systems. The cutoff refers to literature availability rather than manuscript submission or acceptance. No publication after 1 September 2026 should be represented as part of this search unless an additional update is explicitly documented.

Search terms were organized into three concept groups and combined using the Boolean operators AND and OR. The production system group included “hydroponic,” “soilless culture,” and “nutrient solution.” The sensing and monitoring group included “nutrient sensing,” “nutrient monitoring,” “electrical conductivity,” “pH,” “ion-selective electrode,” “ion sensor,” “spectroscopy,” and “IoT.” The control group included “nutrient dosing,” “fertigation,” “feedback control,” “closed-loop control,” “PID,” “model-based control,” and “predictive control.” A representative search expression was (“hydroponic” OR “soilless culture” OR “nutrient solution”) AND (“nutrient sensing” OR “nutrient monitoring” OR “ion-selective electrode” OR spectroscopy OR EC OR pH) AND (“nutrient dosing” OR “feedback control” OR “closed-loop control” OR PID OR “model-based control” OR IoT). The syntax was adapted to the search fields and operators supported by each database. For reproducibility, the database-specific field codes, complete executed search strings, filters, result counts, and export dates should be retained alongside the screening record; the representative expression is not a substitute for these records.

Coverage is a limitation of this review. Two primary databases and supplementary citation tracking may underrepresent engineering conference proceedings, sensor development studies, regional journals, and commercial deployment documentation. The three-concept search may also omit sensing studies that do not mention control. A broader update should separate sensing and control searches and add appropriate sources such as IEEE Xplore and CAB Abstracts, with each additional source and its records reported. Until that update is executed and screened, the review should be interpreted as a structured critical synthesis rather than an exhaustive census of the field.

Studies were eligible when they examined nutrient solution sensing, sensor calibration, monitoring, data acquisition, nutrient dosing, control algorithms, actuation, feedback, or integrated monitoring and control in hydroponic or related soilless cultivation. Studies focused exclusively on crop nutrition, fertilizer response, plant physiology, or soil-based nutrient management were excluded when they did not include a relevant sensing or control component. Peer-reviewed journal articles formed the principal evidence base. Conference papers, book chapters, and review articles were retained only when they provided essential methodological, foundational, or technological information directly relevant to the review objectives.

The searches identified 301 records. After 65 duplicate records were removed by comparing titles, authors, publication years, and DOI information, 236 unique records remained for title and abstract screening. Of these, 148 records were excluded because they did not meet the defined scope, and 88 full-text articles were assessed for eligibility. A further 27 full-text articles were excluded because they lacked a relevant hydroponic or soilless context, did not address a sensing or control component, or did not provide sufficient methodological or technological information for the planned synthesis. The final evidence base therefore comprised 61 studies. Figure 1 reconstructs these aggregate counts. Database-specific and supplementary source totals, retrieval failures, and reason-specific full-text exclusions are not recoverable from the manuscript alone and require reconciliation with the screening log. The number of cited references need not equal the number of included studies because reporting guidelines and background sources may also be cited.

Screening reproducibility also depends on documenting who screened each stage, whether decisions were independent, how disagreements were resolved, and whether eligibility decisions were checked against a common set of criteria. Agreement should be calculated from the reviewers’ initial decisions before adjudication. For two reviewers, Cohen’s κ = (p_o_ − p_e_)/(1 − p_e_), where p_o_ is observed agreement and p_e_ is agreement expected from the marginal decision frequencies. A κ value cannot be calculated retrospectively from aggregate inclusion counts.

Information was extracted from each included study using a structured evidence matrix. The extracted items comprised the target variable or nutrient ion, sensing principle, operating or measurement range, calibration procedure, analytical performance, interference effects, validation conditions, controller type, actuator configuration, feedback mechanism, operational limitations, and reported level of system integration. Monitoring technologies were classified as conventional physicochemical, ion-specific electrochemical, optical or spectroscopic, multi-sensor, or IoT-enabled approaches. Control approaches were classified as threshold or on–off, PI/PID or rule-based, ion-specific, and model-based or predictive control.

The evidence was synthesized through structured qualitative comparison rather than quantitative meta-analysis. Statistical pooling was not considered appropriate because the included studies differed substantially in sensor type, nutrient formulation, crop, experimental scale, validation duration, and reported performance metrics. The technologies were compared in terms of analytical performance, temporal stability, operational robustness, extent of control-loop integration, validation level, reported limitations, and suitability for continuous greenhouse operation. The readiness categories used in this review are qualitative comparative descriptors and should not be interpreted as standardized technology readiness levels.

Quantitative findings are separated by endpoint: concentration or ratio tracking, response steps, and cultivation or resource outcomes. The quantitative synthesis presents five illustrative study results, including two additional contextual examples examined during revision. It is not a census of all quantitative studies, and its heterogeneous endpoints are not pooled. Results from overlapping authors, research networks, or shared experimental platforms should not be treated as independent replication without checking study provenance. Original screening decisions and the membership of the 61-study set require author confirmation.

## 3. Integrated Monitoring and Control Framework

The monitoring framework begins with representative sampling or in situ measurement of the nutrient solution at the reservoir, delivery line, drainage return, or root zone. Sensors detect the relevant physical or chemical properties and convert them into electrical or optical signals. These signals are then conditioned and digitized by the data acquisition system. A microcontroller, programmable logic controller (PLC), edge device, or greenhouse computer processes the measurements through calibration, compensation, validity checking, and signal filtering. Monitoring is considered complete once the processed data have been time-stamped, stored, communicated, visualized, and made available for interpretation or subsequent control. This distinguishes monitoring from control because physical adjustment occurs only when a measured condition is compared with a target and the resulting deviation is converted into an actuator command.

Control therefore begins with a validated measurement and a predefined setpoint, acceptable operating range, or model-derived target. Based on the detected deviation, the controller determines the required input, which may include nutrient stock solution, water, acid, base, aeration, or additional circulation. Dosing pumps, solenoid valves, injectors, or circulation units then deliver the required amount. After each adjustment, sufficient time must be allowed for hydraulic mixing and sensor stabilization before a new measurement is accepted. Without this delay, localized concentration gradients can produce misleading readings and lead to repeated or excessive dosing. Feedback monitoring subsequently determines whether the corrective action has achieved the intended target while ensuring that the system remains within predefined safety limits [12,13].

**Figure 2:**
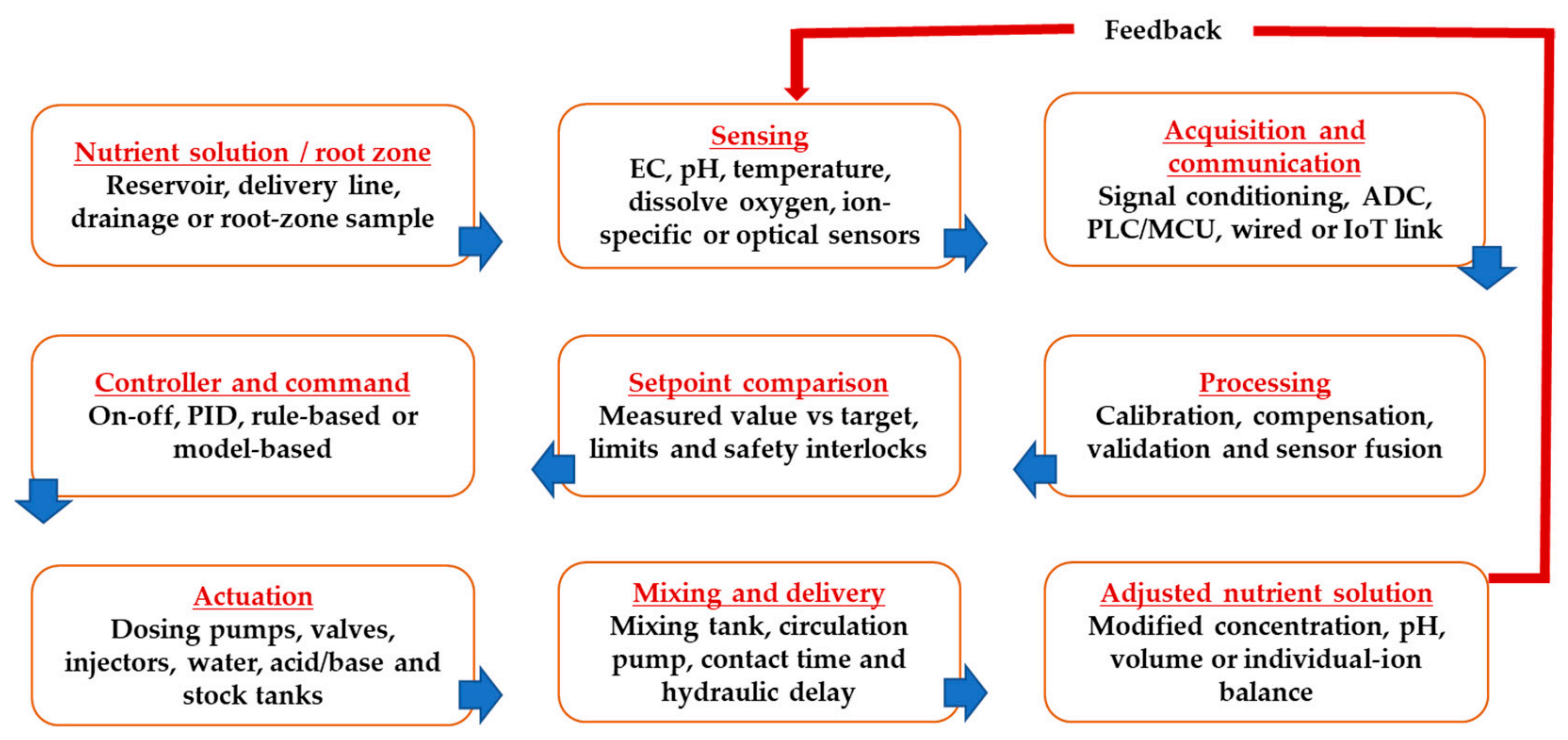
Integrated nutrient monitoring and control framework for hydroponic crop production in a smart greenhouse. Sampling establishes a representative solution, sensing converts the target variable to a signal, acquisition conditions and digitizes the signal, local processing applies calibration and validity checks, the controller compares accepted data with targets, actuators deliver bounded corrective inputs, and mixing and stabilization precede feedback verification. Storage, communication, diagnostics, and safety functions support these modules.

Figure 2 summarizes the integrated monitoring control chain. Open-loop systems apply predetermined nutrient quantities or fixed schedules without using measurements to verify the resulting solution condition. In operator-supervised or human-in-the-loop control, selected variables are monitored and recommendations or alarms are generated, but the operator authorizes the physical adjustment. Closed-loop systems automatically compare validated measurements with targets, execute corrective action, and remeasure the response. Fully automated closed-loop systems additionally coordinate calibration, dosing and mixing sequences, communication recovery, alarm handling, fault detection, and safe shutdown. These control-loop categories should not be confused with hydraulic recirculation: a recirculating hydroponic system may still use manual or open-loop nutrient dosing.

Within this framework, monitoring provides validated information about the nutrient solution. Control uses that information to select a corrective action and verifies the response after dosing and mixing. Sampling location, sample transport delay, mixing volume, actuator capacity, and return flow timing affect the control result [12,19]. Calibration status, invalid data rejection, delivered dose verification, dosing limits, interlocks, and event records must therefore be evaluated across the complete chain.

**Figure 3:**
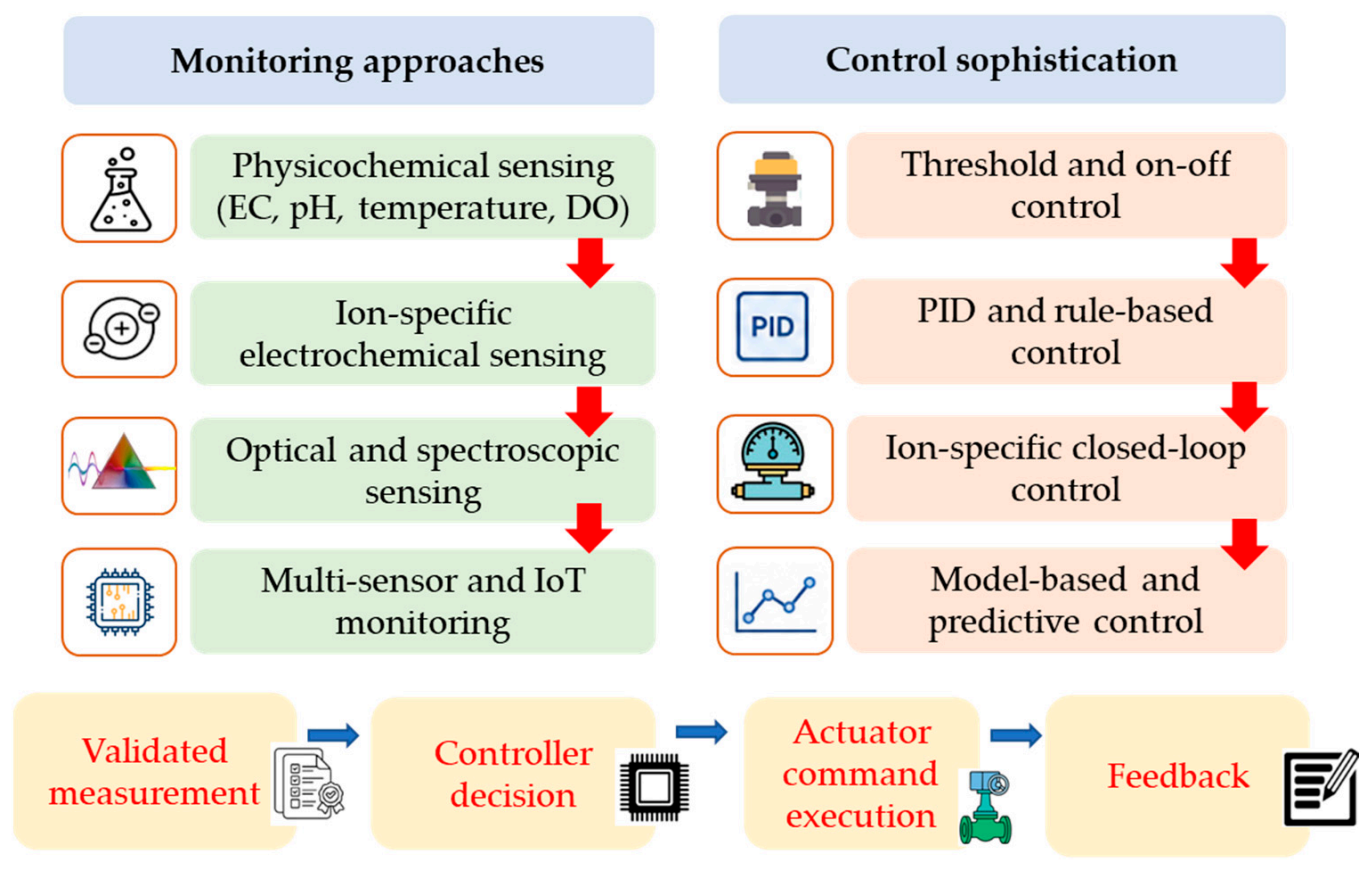
Classification of nutrient monitoring technologies and control approaches retained in this review.

Figure 3 summarizes the principal monitoring and control approaches considered in this review. Monitoring technologies progress from conventional physicochemical sensing to ion-specific, optical, and multi-sensor/IoT-enabled approaches, while control strategies progress from threshold-based operation to PI/PID and rule-based, ion-specific, and model-based/predictive control. The lower sequence emphasizes the complete pathway from validated measurement to controller decision, actuator command execution, and feedback verification.

## 4. Nutrient Monitoring Technologies

Hydroponic nutrient monitoring spans mature bulk solution measurements and emerging ion-specific, spectroscopic, multi-sensor, and connected platforms. Table 1 compares their operating principles, strengths, limitations, and readiness for continuous greenhouse application.

The readiness descriptors in Table 1 summarize the evidence available for application, rather than formal TRLs or a numerical technology certification. Table 2 defines the evidence gates used for interpretation. Assessment should be attached to a specific device and application, not transferred automatically to every sensor sharing the same principle. Validation duration, cultivation environment, calibration/cleaning interval, and feedback integration should be extracted separately. An unreported criterion is “not reported” and does not count as a passed gate. “High” for conventional technologies denotes established routine use; it does not assert that every implementation has been validated by the present review.

For a device-level assessment, record the tested crop and nutrient matrix, deployment duration, calibration and cleaning events, data availability, and verified feedback functions. Apply the Table 2 descriptors only when every criterion in the selected row is supported; otherwise, use the highest fully supported row or “insufficient information.” Duration refers to continuous cultivation exposure, and a full crop cycle refers to the studied crop. Table 1 retains broad technology context rather than certifying specific products. These are review-defined evidence gates, not an externally validated scoring scale.

The greenhouse readiness categories in Table 1 were developed in this review to provide a qualitative indication of the evidence supporting continuous greenhouse application and should not be interpreted as formal Technology Readiness Levels (TRLs). High readiness indicates mature technologies that are routinely applicable in greenhouse operation with conventional calibration and maintenance. Moderate to high refers to established technologies with demonstrated greenhouse integration but with some remaining requirements for site-specific or long-term validation. Moderate indicates technologies that have been demonstrated under greenhouse conditions but still face limitations related to calibration stability, maintenance, or operational robustness. Low to moderate represents technologies supported mainly by laboratory- or pilot-scale studies with limited evidence of sustained continuous operation, whereas prototype to pilot denotes technologies demonstrated primarily as proof-of-concept or short-duration integrated systems. These categories were assigned comparatively based on the validation conditions, operational evidence, maintenance requirements, and level of system integration reported in the cited studies, rather than being adopted directly from an existing standardized readiness framework.

### 4.1. Conventional Physicochemical Monitoring

Electrical conductivity (EC) remains the principal online indicator of overall nutrient solution concentration. In EC measurement, an alternating electrical field is applied between the electrodes of a conductivity cell, and the resulting conductance of the solution is measured. Conductance is subsequently converted to conductivity using the cell constant, with temperature compensation generally being applied because conductivity is temperature dependent. EC can be measured rapidly and readily integrated into automated monitoring and control systems; however, the measured value represents the combined contribution of the concentration and ionic mobility of all dissolved charged species. Consequently, an EC value within the prescribed range does not indicate whether individual nutrients, such as nitrate, potassium, calcium, magnesium, or micronutrients, are maintained within their respective target concentrations. For reliable continuous measurement, the sensor must be calibrated using an appropriate conductivity standard, temperature compensation must be verified, adequate sensor immersion and solution flow must be maintained, and deposits or air bubbles on the conductivity cell must be prevented or removed [9,13].

The pH of a nutrient solution is commonly measured using a glass electrode paired with a reference electrode, although equivalent solid-state sensors can also be used. The potential difference between the sensing and reference electrodes reflects hydrogen-ion activity and is converted into a pH value after calibration with standard buffer solutions. Because pH sensors can provide continuous measurements, they are readily integrated with acid- and base-dosing systems and are widely used to maintain nutrient solutions within a desired pH range.

Reliable pH measurement, however, requires careful sensor maintenance and operation. Measurement accuracy may decline because of reference junction contamination, electrode aging, temperature fluctuations, low ionic strength, surface deposits, electrical interference, or poor grounding. Regular two- or three-point calibration, routine cleaning, proper electrode storage, and periodic comparison with an independent reference measurement are therefore important for maintaining measurement reliability. The timing of measurements is equally important in automated control. After nutrients, acid, or base are added, the solution requires sufficient time for mixing and for the sensor response to stabilize. Accepting a new pH reading too soon can result in an unrepresentative measurement and unnecessary additional dosing. An appropriate mixing and stabilization interval should therefore be incorporated into the control sequence before the next dosing decision is made [13].

Solution temperature and dissolved oxygen (DO) complement EC and pH monitoring. Thermistors or resistance temperature detectors measure solution temperature, which indicates root zone thermal conditions and supports compensation of conductivity and other electrochemical signals. Electrochemical or optical probes measure DO, an important root zone variable in deep-water and warm recirculating systems because inadequate oxygen can impair root function. Optical probes avoid oxygen consumption during measurement but still require sensing cap inspection and fouling control; electrochemical probes additionally require membrane and electrolyte maintenance. These variables improve interpretation of solution status but do not directly quantify nutrient ions [9,21,22].

### 4.2. Ion-Specific Nutrient Sensing Technologies

#### 4.2.1. Ion-Selective Electrodes

Ion-selective electrodes (ISEs) employ an ion-selective membrane through which preferential interaction with a target ion generates an electrical potential relative to a reference electrode. According to potentiometric theory, the electrode potential varies approximately as a logarithmic function of ion activity. Accordingly, calibration is generally performed using standard solutions that span the expected operating range. Ion-selective membranes are available for several agriculturally important ions, including nitrate (NO_3_^−^), potassium (K^+^), calcium (Ca^2+^), and ammonium (NH_4_^+^). However, the measured response can be affected by membrane selectivity, ionic strength, pH, temperature, and the presence of competing ions. In complex hydroponic nutrient solutions, ion activity rather than an interference-free ion concentration is fundamentally measured. Therefore, matrix-appropriate calibration and periodic validation against an established analytical reference method are required for reliable quantitative application [8,9].

Cho et al. [8] demonstrated the feasibility of greenhouse-oriented ion-specific monitoring using an automated calibration and measurement system for NO_3_^−^, K^+^, and Ca^2+^ in nutrient solutions used for paprika cultivation. When the ISE measurements were compared with ion chromatography, a regression slope of 0.99 and an R^2^ value of 0.99 were obtained for NO_3_^−^. For K^+^ and Ca^2+^, regression slopes of 1.17 and 0.75 and R^2^ values of 0.99 and 0.97, respectively, were reported. Across the evaluated hydroponic samples, root mean square errors below 20 mg L^−1^ were reported within the tested concentration range of 40–1200 mg L^−1^. These results demonstrated the potential of automated ISE-based systems for on-site nutrient monitoring after appropriate calibration factor correction. However, the deviation of the regression slopes from unity, particularly for K^+^ and Ca^2+^, also indicated that a high coefficient of determination alone should not be interpreted as evidence of negligible analytical bias. The 40–1200 mg L^−1^ interval summarizes the reported hydroponic validation range and should not be treated as an ion-specific nitrate range or extrapolated outside of the tested nutrient matrices. The regression sample count and separate concentration ranges for each ion are not specified in the extracted evidence and therefore cannot be inferred from R^2^.

Long-term ISE use is constrained by membrane aging, reference electrode drift, fouling, bubbles, junction blockage, and changes in solution composition. Automated rinsing, calibration with standards, redundant sensing, temperature compensation, and plausibility checks can reduce these effects, although each measure adds maintenance and potential failure points. Phosphate is especially challenging: cobalt-based electrodes have quantified phosphate in hydroponic solutions, but response depends on electrode conditioning and solution properties, including pH, temperature, dissolved oxygen, and interfering species. Reported systems therefore support controlled or intermittent phosphate analysis more strongly than unattended crop cycle monitoring [24,28,29].

Cross-study benchmarking remains difficult because analytical accuracy is reported more consistently than response time, recalibration frequency, long-term drift, or crop cycle stability. Cho et al. [8] reported strong short-term analytical agreement but also ion-dependent calibration bias, whereas Wu et al. [23] demonstrated element-specific nutrient monitoring over a two-week indoor and greenhouse cultivation period. Together, these studies highlight the need to report analytical error, calibration bias, validation duration, and operating environment alongside correlation-based metrics. Table 3 summarizes the operational evidence and unresolved maintenance information for continuous nutrient sensing.

Maintenance should be reported as events per sensor month, labor minutes per event, consumables per month, replacement frequency, and downtime attributable to each intervention. A useful annualized cost separates replacement purchase cost from calibration standards, reagents, cleaning materials, labor, and outage costs. None of these quantities should be assigned a generic numerical value without device- and site-specific evidence.

A commercial calibration example makes the logistics concrete: Bluelab specifies initial pH calibration with pH 7 and pH 4 or 10 buffers and gives a 30-day recalibration reminder for its Pro Controller Wi-Fi [32]. For annual planning, a 30-day schedule corresponds to approximately 13 events per probe year. If an event takes t minutes, scheduled calibration labor is approximately 13t/60 h per probe year before cleaning, troubleshooting, or replacement. Buffer consumption, intervention time, and control downtime must be measured at the deployment site. This manufacturer schedule is a device-specific operating recommendation, not measured evidence that all pH probes remain accurate for 30 days.

#### 4.2.2. ISFETs, Solid Contact Sensors, and Other Electrochemical Approaches

Ion-sensitive field effect transistors and solid-contact ISEs are distinct miniaturized electrochemical platforms. Field effect devices convert ion-dependent surface potential into transistor current changes, whereas solid-contact ISEs replace the internal filling solution with an ion-to-electron transducer beneath the selective membrane. Both can support compact arrays, small sample volumes, and embedded electronics. A field-effect sensor array has detected phosphate, nitrate, and potassium in nutrient solution, and an IoT-interfaced solid contact array has monitored K^+^, NO_3_^−^, and NH_4_^+^ during two-week indoor and greenhouse cultivation. More recently, screen-printed biochar-derived graphene solid contact ISEs achieved near-Nernstian responses for K^+^, Na^+^, NH_4_^+^, Ca^2+^, Mg^2+^, and NO_3_^−^ and were applied to monitor potassium depletion in hydroponic solution [31]. Nevertheless, both platforms still require stable reference potentials, matrix-appropriate calibration, temperature control, and fouling management; miniaturization alone does not establish maintenance-free greenhouse operation [23,33].

Other electrochemical approaches include amperometric, voltametric, impedimetric, and flow injection potentiometric measurements. Their selectivity and sensitivity depend on the target reaction, electrode material, sample conditioning, and nutrient matrix. For example, flow injection potentiometry with a cobalt wire electrode has been used for phosphate determination in hydroponic solution, but electrode surface condition and matrix effects remain operational constraints. Such methods are best treated as specialized analytical modules unless sampling, cleaning, calibration, and quality assurance are automated and validated for prolonged use [9,28]. Table 4 compares the ion-specific sensing technologies discussed in this section.

### 4.3. Optical and Spectroscopic Nutrient Monitoring

Optical and spectroscopic methods estimate nutrient concentrations by measuring how light interacts with the nutrient solution, including wavelength-dependent absorption, reflectance, emission, or color development. UV-visible absorption can target nitrate in suitable spectral regions, while colorimetric methods convert a selective chemical reaction into an optical signal. Reagent-free absorption methods are particularly attractive for continuous or online monitoring because they do not require chemical reagents and can simplify routine operation; reagent-based methods require controlled sampling, reagent delivery, reaction time, waste handling, and blank or standard measurements. Optical measurements are also sensitive to the composition and physical condition of the nutrient solution. Turbidity, naturally or chemically colored compounds, air bubbles, surface deposits, overlapping spectral features, and differences in fertilizer formulation can all influence the measured signal. As a result, reliable application requires calibration that reflects the actual nutrient solution matrix, together with regular cleaning and maintenance of the optical path. Therefore, the suitability of optical sensing for automated hydroponic monitoring depends not only on analytical sensitivity but also on its ability to maintain stable performance under changing greenhouse operating conditions [9,24,25].

Near-infrared and multispectral sensing systems generally rely on multivariate calibration rather than a single unique absorption feature associated with an individual nutrient. Stevens et al. [34] evaluated a waterproofed IoT-enabled spectroscopic sensor in a microscale hydroponic system. A multiple linear regression model with an R^2^ value of 0.997 was reported for prediction of nutrient addition volume, while a correlation coefficient of 0.77 was obtained between the predicted nutrient quantity and conventional EC measurements during system deployment. These results demonstrated the feasibility of low-cost spectroscopic integration into hydroponic monitoring systems. However, the reported performance should not be interpreted as general validation of individual-ion quantification across different nutrient formulations or commercial greenhouse conditions.

Recent studies illustrate the trade-off between analytical specificity and operational simplicity. Han et al. [24] used a portable molybdenum-based colorimetric method for phosphate, demonstrating a compact analytical workflow but retaining reagent and incubation requirements. Silva et al. [25] evaluated reagent-free spectroscopy for N, P, and K estimation and found that multivariate modeling could extract nutrient information, although phosphorus and potassium estimates were more affected by spectral interference than nitrogen estimates. Accordingly, greenhouse deployment should be judged not only by calibration statistics but also by sampling representativeness, model transferability, cleaning needs, environmental protection, and sustained performance in changing nutrient matrices.

### 4.4. Sensor Arrays, Multi-Sensor Platforms, and Electronic Sensing Systems

Hydroponic nutrient solutions contain many interacting ions, and no single measurement can fully describe overall ionic strength, individual nutrient concentrations, temperature effects, hydraulic conditions, and sensor performance. For this reason, multi-sensor platforms commonly integrate measurements of electrical conductivity (EC), pH, temperature, dissolved oxygen (DO), individual ions, flow, water level, and drainage characteristics. Temperature measurements can be shared across sensors for compensation, while EC provides a useful indication of the overall ionic status of the solution against which ion-specific estimates can be evaluated. Combining multiple signals also makes it possible to apply data validity checks. For example, a sudden change in the estimated concentration of an ion may be flagged as potentially unreliable when it is not accompanied by a corresponding dosing, dilution, or hydraulic event [9,12,23].

Electronic tongues extend this concept by using arrays of partially selective sensors and interpreting their combined response patterns with multivariate models, rather than requiring each sensing element to be completely selective. Gutiérrez et al. [26] demonstrated that this approach can reduce cross-sensitivity when the calibration dataset adequately represents the nutrient solution conditions encountered during operation. However, sensor aging, changes in fertilizer formulation, temperature fluctuations, and operation outside of the original calibration range can gradually reduce model accuracy. Automated sampling, standard addition, and rinsing can improve measurement repeatability, but miniaturized fluidic systems remain susceptible to precipitation, air bubbles, biofilm formation, and blockage. Sensor fusion therefore does not eliminate the selectivity challenge; instead, part of that challenge shifts toward calibration management, data quality assessment, and routine system maintenance. Tuan et al. [35] further combined automated multivariate standard addition with a deep kernel learning model for on-site determination of Ca^2+^, phosphate, and Mg^2+^ in a lettuce hydroponic system, illustrating the potential of model-assisted multi-ion diagnostics while retaining dependence on automated sampling and matrix-specific calibration.

### 4.5. IoT-Enabled and Real-Time Nutrient Monitoring Systems

IoT-enabled nutrient monitoring connects sensors and actuators in the greenhouse with local data acquisition and processing units and, when required, with remote servers or cloud platforms. For nutrient management, however, connectivity alone does not determine system performance. The more important function is the conversion of raw sensor signals into reliable, time-stamped measurements through calibration, compensation, range checking, filtering, and assessment of sensor status. These tasks may be performed by microcontrollers, PLCs, edge computers, or greenhouse control computers before the data are transmitted for storage, visualization, or higher-level analysis [36,37].

Real-time monitoring should therefore be considered as the complete pathway from measurement acquisition to validated, time-synchronized information delivery for interpretation or subsequent control. Information such as sampling time, measurement interval, calibration status, sensor identity, missing data, manual interventions, and actuator events should be recorded together. This allows changes in nutrient concentration to be interpreted in relation to dosing, dilution, irrigation, circulation, or drainage events and improves the traceability of both measurements and control actions [9,38,39].

A clear separation between local and cloud-based functions is particularly important when the system performs autonomous dosing. Cloud services are well-suited to long-term data storage, visualization, and advanced analytics, whereas essential control functions should remain available locally. Measurement validation, dosing limits, circulation control, alarms, and safe shutdown procedures should therefore continue to operate even when network communication is interrupted. Local data buffering and recovery mechanisms are also needed to preserve measurement and actuator records during temporary communication failures [36,37].

Automation, including robotic handling where implemented, should be evaluated against environmental objectives as well as operational reliability. Reducing the impact of the technological production infrastructure on the biosphere requires recording freshwater input, nutrient recovery and discharge, and power consumption alongside crop output. These records can support resource budgets and constrained control objectives; connectivity or a higher degree of automation alone does not demonstrate sustainable performance. Nutrient and water balances provide a basis for such assessment [40].

Stevens et al. [30] observed electrical interference from an analog water-level sensor producing unreliable EC and pH readings and loss of retained calibration. Removing the level sensor did not restore reliable calibration, so EC and pH were measured manually during the experiment. This case illustrates a failure of measurement integration that restricted automated feedback, rather than a wireless link failure. It supports testing grounding, isolation, power stability, and sensor compatibility before autonomous dosing.

Tatas et al. [27] operated two control units and four environmental nodes for approximately 1000 h and recorded sensor drift/outliers and occasional GPRS transmission failures. Retries corrected many communication errors, but one interruption required intervention with the service provider. These observations establish that remote data delivery can fail even while a local platform remains operational; they do not quantify nutrient control error during outages. For autonomous dosing, the design implication is to retain local measurement validation and dose limits, buffer records, flag stale data, and inhibit commands that depend on unavailable measurements.

Communication technology should be selected according to greenhouse size, transmission range, available power, bandwidth requirements, and acceptable latency. Wi-Fi is common in experimental systems, whereas Zigbee, LoRa/LoRaWAN, Bluetooth Low Energy, and cellular communication provide different trade-offs in range, power consumption, and data capacity. For scalable nutrient management systems, interoperability is ultimately more important than the popularity of a particular communication platform. Sensors, dosing equipment, PLCs, edge devices, and greenhouse software should be able to exchange not only measurement values but also timestamps, calibration status, data quality flags, actuator states, and alarm information in a consistent format [17]. Cybersecurity also becomes a process safety issue when remote communication can activate fertilizer, acid, base, water, or circulation equipment. Such systems should therefore include authenticated access, appropriate authorization levels, protected communication, command and event logging, and local interlocks that remain effective if remote connectivity is lost. Performance evaluation should extend beyond basic connectivity and include measurement availability, missing data rate, communication latency, recovery from network or power failure, calibration traceability, actuator command traceability, system uptime, and the proportion of invalid measurements rejected before they are used for control [17,41]. A quantitative LoRa versus Wi-Fi ranking for metal-framed greenhouses cannot be inferred from protocol specifications or studies with different antenna heights, structures, crops, or payloads. A matched deployment comparison should report the packet delivery ratio, end-to-end latency and jitter, longest outage, power use, recovery time, and resulting control error under the same greenhouse geometry. The cases above provide failure evidence but not such a matched radio comparison.

## 5. Performance Evaluation of Monitoring Technologies

A rigorous assessment of monitoring technologies requires evaluation beyond short-term sensor accuracy. For continuous greenhouse monitoring and control, performance can be assessed across four linked levels: analytical, temporal, operational, and system/control performance (Table 5). The proposed framework represents a progression from intrinsic measurement quality to dynamic behavior, long-term deploy ability, and eventual suitability for closed-loop control. This distinction is important because satisfactory calibration performance alone does not ensure reliable measurements under realistic nutrient solution conditions or during prolonged cultivation [8,23,42].

Analytical performance describes whether a monitoring technology can provide measurements of sufficient quality for its intended use. Evaluation should therefore consider bias, prediction error such as RMSE, sensitivity, selectivity, detection limits, repeatability, and measurement uncertainty. For ion-selective electrodes, these characteristics should be assessed under clearly defined measurement conditions because sensitivity, selectivity, detection limits, and response behavior can vary with the evaluation protocol [42]. Comparison with an established reference method is also important, as good calibration statistics in standard solutions do not necessarily indicate reliable performance in complex nutrient solutions. Validation should therefore cover concentration ranges and solution compositions that reflect actual cultivation conditions [8,23,29].

Temporal performance refers to how quickly and consistently a sensor responds over time. Important indicators include response time, recovery time, stabilization time, drift, and recalibration interval. These characteristics should be considered separately from analytical accuracy because a sensor may perform well under stable conditions but still respond too slowly or drift excessively during continuous operation. For feedback control, response and stabilization times should also be evaluated in relation to the dynamics of the controlled process [42].

Operational performance concerns whether a sensor can maintain acceptable performance under realistic greenhouse conditions. Relevant factors include fouling resistance, cleaning frequency, consumable use, maintenance effort, sensor lifetime, and robustness to changes in temperature, flow, air bubbles, and nutrient solution composition. Matrix effects and interfering ions are particularly important for ion-selective sensing because they can alter sensor response and reduce prediction accuracy [8,29]. Several hydroponic monitoring systems have therefore incorporated multi-ion calibration, normalization, automatic sampling, and comparison with reference methods to improve reliability during practical greenhouse operation [8,23,29]. Operational studies should also report how often maintenance is required, how long it takes, and how it affects data availability.

System/control performance considers whether the monitoring technology can function reliably within an integrated decision or feedback control system. Relevant indicators include missing data rate, uptime, communication latency, synchronization between sensors and actuators, fault detection, and rejection of invalid measurements before they influence control decisions. These aspects become more important as monitoring systems include automated sampling, embedded electronics, wireless communication, cloud services, and nutrient management algorithms [23]. When sensor outputs are used directly for dosing, evaluation should also include actuator response, settling time, overshoot, and overall control stability. Ion-specific nutrient management studies have shown that the complete sensing–decision–dosing sequence must be validated, rather than assessing sensor accuracy alone [11].

Validation should therefore progress from controlled laboratory testing to increasingly realistic operating conditions. Initial calibration should establish the basic analytical characteristics of the sensor [42]. This should be followed by validation in representative nutrient solution matrices and comparison with established analytical methods [29]. The next stage should evaluate longer-term stability during cultivation, including drift, recalibration needs, data continuity, and performance under actual crop production conditions. Studies conducted during paprika cultivation and multiweek hydroponic experiments illustrate the value of evaluating sensors beyond laboratory-prepared solutions [8,23]. Maintenance requirements, fault behavior, and recovery from abnormal conditions should then be assessed before the technology is used in closed-loop control. For automated systems, the final stage should validate the complete sensor–controller–actuator chain under representative operating conditions [11]. Table 5 links each level to a technical case and identifies the boundary of the evidence. Passing an analytical check cannot compensate for an untested maintenance burden or incomplete control verification.

Studies that compare sensor measurements with established analytical methods under realistic hydroponic conditions provide stronger evidence of practical applicability than calibration curves alone. Cho et al. [8], for example, compared NO_3_^−^, K^+^, and Ca^2+^ measurements from an automated ISE system with standard analytical measurements using nutrient solutions collected during paprika cultivation. Wu et al. [23] combined laboratory comparison with ion chromatography and commercial electrodes with subsequent monitoring during hydroponic cultivation. Jung et al. [29] further showed that testing in representative paprika nutrient solutions can reveal limitations that may not be apparent under simplified calibration conditions.

## 6. Nutrient Control and Actuation Technologies

Table 6 consolidates operating scenarios, dynamic response, reported control errors, tuning requirements, and limitations. Numerical results are retained in their original variables and units, and they cannot be ranked as equivalent outcomes. No matched set of response time, steady-state error, disturbance rejection, and tuning effort was available across all five strategies. Table 6 and Table 7 therefore distinguish reported evidence from metrics still required for a fair comparison.

### 6.1. Dosing and Physical Actuation Hardware

Physical nutrient control relies on dosing pumps, peristaltic pumps, injectors, solenoid valves, stock solution tanks, water dilution valves, circulation pumps, and the associated tubing and manifolds. Even when the controller calculates the correct dose, the amount actually delivered may differ because pump output is influenced by the suction head, back pressure, tubing condition, and hydraulic resistance. Peristaltic pump flow can vary with operating conditions, while fertigation injectors may lose accuracy when particles restrict flow or alter line pressure [52,53].

For this reason, dosing devices should be calibrated under their installed operating conditions rather than relying only on nominal pump specifications. Stock solution concentration and level, pump runtime or delivered volume, valve position, dilution water flow, and circulation status should be recorded whenever possible. Without such verification, blocked lines, depleted stock tanks, valve leakage, pump wear, or precipitation can cause a correct control decision to produce an incorrect nutrient adjustment [13,54].

Mixing is also part of the actuation process because the added concentrate must be sufficiently homogenized before a new measurement is accepted. Local safety functions should therefore include limits on single and cumulative dosing, minimum intervals between additions, confirmation of circulation, low-stock alarms, actuator status checks, leak detection, and a predefined safe shutdown condition. In this way, dosing accuracy and actuator verification become integral components of closed-loop performance rather than secondary hardware considerations.

### 6.2. Threshold and On–Off Control

Threshold and on–off control compare continuously or periodically measured EC and pH with acceptable bands and actuates nutrient, water, acid, or base delivery when a limit is crossed. The approach is simple and operationally robust, but overshoot and oscillation occur when dose resolution, hysteresis, reservoir volume, and transport/mixing delays are poorly matched. EC control maintains a bulk concentration envelope, whereas pH control maintains acid–base condition; neither alone guarantees individual ion balance [13,44,55].

**Figure 4:**
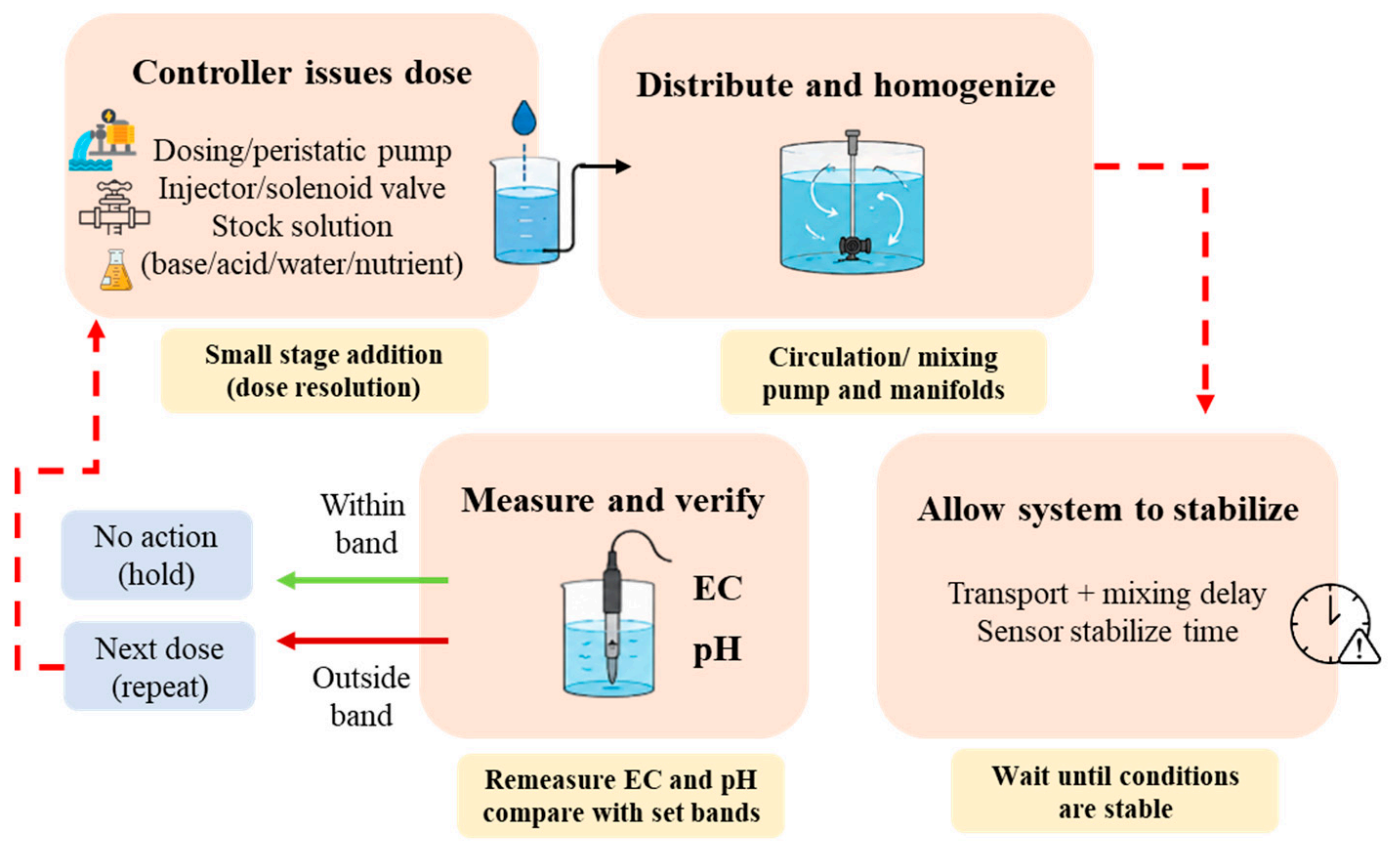
Threshold-based nutrient control cycle and key performance layers for closed-loop hydroponic management.

Small staged additions reduce overshoot because each actuation is followed by mixing, sensor stabilization, and remeasurement before another command is issued. In the multichannel system reported by Sun et al. [43], accounting for transport and mixing behavior supported EC control within approximately 0.05 mS cm^−1^. Four performance layers should be distinguished: controller output accuracy, physical dosing accuracy, chemical mixing and transport dynamics, and measurement accuracy. Figure 4 summarizes this control cycle and the four performance layers.

The principal limitation of EC feedback is chemical non-specificity. Different ionic mixtures can have similar EC values, and selective crop uptake can deplete one nutrient while others accumulate. Periodic laboratory analysis, mass balance estimates, or selected ion-specific measurements can therefore complement EC during prolonged recirculation [56,57]. Gang et al. [49] combined EC with ISE measurements and an ion-equivalent concentration ratio algorithm; the final target ratio error was 5.9%, representing a reported 58% improvement over EC-only replenishment. This hybrid approach uses EC as a rapid bulk signal while selected ion measurements correct compositional drift.

### 6.3. PI/PID and Rule-Based Control

PI/PID control modulates dosing according to present and accumulated error and, for PID, the error rate of change. Its parameters must reflect sensor response, pump flow, hydraulic dead volume, reservoir size, and chemical nonlinearity; system-specific identification and bounded actuator outputs are therefore essential, particularly for concentrated acid or fertilizer delivery [13,45].

Rule-based and fuzzy controllers can encode staged dosing, nonlinear operating knowledge, mutual exclusion of acid and base additions, and other safety constraints that are difficult to represent with a single linear controller. Their main limitation is transferability: rule sets and membership functions developed for one crop, reservoir size, or dosing configuration may require re-identification in another system [47]. Vincentdo and Surantha [48] applied an adaptive neuro-fuzzy inference system (ANFIS) to nutrient film-technique monitoring and control, providing an example of data-driven fuzzy control integrated with a hydroponic platform.

### 6.4. Ion-Specific Nutrient Control

Ion-specific control extends conventional EC-based nutrient management by using the measured concentrations of individual ions to guide fertilizer replenishment. Instead of regulating only the overall ionic strength of the nutrient solution, the controller identifies the deficit of each target nutrient and calculates the required addition of available fertilizer stock solutions. This approach is particularly useful in closed hydroponic systems where unequal nutrient uptake and repeated recirculation can gradually change the balance among individual ions even when EC remains within the target range [11,19,49].

Cho et al. [11] demonstrated ion-specific control by combining ion-selective electrode measurements with a decision-tree-based dosing algorithm that determined fertilizer additions for five major nutrient ions while accounting for the contribution of multiple ions by individual fertilizer salts. During a five-step replenishment experiment, mean relative errors were 8.0 ± 11.0% for NO_3_^−^, 7.9 ± 2.1% for K^+^, 10.6 ± 8.0% for Ca^2+^, and 4.2 ± 3.7% for solution volume. Some replenishment steps resulted in over- or undersupply, indicating that dosing performance depends not only on the control algorithm but also sensor accuracy, pump performance, mixing, and the nutrient composition remaining from the previous step [11]. These results support a closed-loop strategy in which ion concentrations are remeasured after each dosing action and subsequent additions are recalculated from the updated nutrient status. The five-step experiment is a single-study feasibility result, not a population estimate of ion-specific control accuracy. The reported dispersion and step-dependent dosing deviations should be retained; the three mean errors should not be repeated as expected commercial performance.

For model-based control, the evolution of nutrient concentration can be represented using a discrete mass balance. Mass balance principles provide a physically consistent basis for describing nutrient dynamics in closed hydroponic systems because changes in solution concentration arise from fertilizer addition, nutrient inflow and outflow, plant uptake, other nutrient losses, and changes in solution volume [40]. A generalized representation for nutrient (i) over one control interval is

(1) $$C_{i,t+1}=\frac{C_{i,t}V_{t}+\sum_{j=1}^{m}S_{j,t}D_{i,j}+C_{i,\mathrm{in},t}V_{\mathrm{in},t}-C_{i,\mathrm{out},t}V_{\mathrm{out},t}-U_{i,t}-L_{i,t}}{V_{t+1}}$$

In Equation (1), *i* indexes nutrient ions, *j* indexes fertilizer stocks, *m* is the number of stocks, and *t* and *t* + 1 denote successive sampling boundaries separated by a control interval Δ*t*. *C_i_*_,*t*_ and *C_i_*_,*t*+1_ are solution concentrations (mg L^−1^), and *V_t_* and *V_t_*_+1_ are solution volumes (L). *S_j_*_,*t*_ is stock volume added during Δ*t* (L), and *D_i_*_,*j*_ is the concentration of ion *i* in stock *j* (mg L^−1^). *C_i_*_,in,*t*_ and *C_i_*_,out,*t*_ are effective inflow and outflow concentrations (mg L^−1^); *V*_in,*t*_ and *V*_out,*t*_ are corresponding volumes transferred during Δ*t* (L). *U_i_*_,*t*_ is uptake mass and *L_i_*_,*t*_ is other loss mass during Δ*t* (mg), not rates. Each numerator term therefore has units of mg, and division by *V_t_*_+1_ gives mg L^−1^. For a molar formulation, all concentration and mass terms must be converted consistently.

The balance assumes a well-mixed control volume, additive dissolved nutrient inputs, and consistent chemical reporting bases. Effective inflow/outflow concentrations must represent the transferred solution; precipitation, irreversible retention, or unmodeled sinks enter the loss term. *V_t_*_+1_ must be measured or calculated from a separate water balance that includes stock addition, make-up water, discharge, evapotranspiration, and any sampling loss. Inflow accounting must exclude stocks already represented by Σ*S_j_*_,*t*_*D_i_*_,*j*_ to avoid double counting.

Earlier replenishment models addressed recycled greenhouse effluents and individual nutrient inputs [58]. They provide methodological context for nutrient balance calculations, while operational comparison still requires common cultivation conditions and performance metrics.

Equation (1) illustrates an important feature of ion-specific nutrient control: the controller adjusts fertilizer sources, while the regulated variables are the concentrations of individual nutrient ions. These variables are inherently linked because most fertilizer salts supply more than one ion. For example, adding Ca(NO_3_)_2_ to correct a Ca^2+^ deficiency also increases NO_3_^−^, while KNO_3_ contributes both K^+^ and NO_3_^−^. Correcting each ion independently may therefore lead to excessive accumulation of associated ions. Cho et al. [11] addressed this problem using a decision tree approach that compared alternative fertilizer combinations and selected additions that minimized unnecessary nutrient supply.

Recent studies have extended this concept toward multivariable and adaptive nutrient control. Kim et al. [19] developed a closed hydroponic management system in which ISE measurements of NO_3_^−^, K^+^, and Ca^2+^ from multiple water sources were used to calculate replenishment through eight fertilizer stock solutions. The system was evaluated during long-term lettuce cultivation in a closed nutrient film technique system. Gang et al. [49] subsequently introduced an adaptive approach in which measured NO_3_^−^, K^+^, and Ca^2+^ concentrations were converted to ion-equivalent concentration ratios and used as feedback to adjust fertilizer composition. Nonlinear programming was then used to determine the required fertilizer volumes according to the ionic condition of the recycled nutrient solution [19,49].

### 6.5. Model-Based and Predictive Control

Model-based dosing extends the coupled formulation problem across time. Opti-Dose uses finite-horizon optimal control to coordinate daily additions from six fertilizer sources while maintaining N, P, K, Ca, Mg, and S within crop-specific adequacy ranges and accounting for water and resource use. Predictive optimization can therefore coordinate multiple nutrients and constraints more systematically than independent threshold rules, but operational value depends on reliable state estimates, parameter identification, actuator calibration, and validation outside of simulation [50].

Nutrient targets should be parameterized for the plant species, cultivar, and developmental stage, rather than being treated as a universal formulation. A proposed integrated controller would combine validated nutritional requirements with water availability, nutrient losses, and plant health information. Mineral nutrition can modify disease resistance or susceptibility, but its effects depend on the host–pathogen interaction and nutrient status [59]. Nutrient solution measurements should therefore complement crop inspection, pathogen surveillance, and sanitation decisions. They cannot independently diagnose disease or establish that pesticides are unnecessary. Changes to fertilization or cultivation practices require crop-specific validation within the complete production system [60].

Emerging approaches combine adaptive control, sensor fusion, digital twins, edge computing, and machine-learning-assisted calibration. Near-term applications are strongest where these methods support drift compensation, anomaly detection, mixing dynamic estimation, and constrained dose selection rather than replacing physical feedback. Model-predictive and data-driven controllers should therefore operate within explicit concentration, dose, and equipment constraints [16,50,51,61].

## 7. Safety Constraints and Feedback Verification

Closed-loop nutrient control should be considered verified only when both the process response and the actuator action are checked. Essential safeguards include measurement validity checks, maximum dose and rate limits, confirmation of circulation, actuator state or delivered flow verification, abnormal response detection, manual override, and predefined fail-safe states. Computational sophistication cannot compensate for unverified sensors, uncalibrated actuators, or inadequate hydraulic mixing; autonomous control therefore requires supervisory safety logic independent of the main optimization algorithm [62,63].

Table 7 separates five illustrative results by controlled variable, endpoint, and validation context. Agustian et al. [46] report discrete response steps for fuzzy pH/TDS control; Bhat et al. [10] include manual and commercial dosing comparators and report cultivation and water use outcomes. These independent examples broaden the quantitative context, but their metrics cannot validate the per-ion errors in Cho et al. [11]. The five-step ion-specific experiment and the hybrid ratio control study remain system-specific demonstrations. Matched setpoints, nutrient matrices, hydraulic volumes, disturbance tests, and error definitions would be required to compare controller accuracy.

## 8. Integrated Nutrient Monitoring Control Systems

The practical value of an integrated monitoring and control architecture depends on how completely the measurement–decision–actuation chain is connected and verified. Systems that only collect or transmit sensor data are more appropriately regarded as monitoring platforms, whereas operator-supervised systems provide decision support but still require human approval before corrective action is taken. In a closed-loop system, measurements are used directly to initiate corrective actions, after which the resulting condition is measured again to confirm the system response. A fully automated closed-loop architecture extends this concept further by incorporating functions such as automatic calibration, verification of actuator operation, alarm management, communication recovery, and fail-safe responses. Distinguishing systems according to these functional levels provides a more meaningful assessment of automation than simply classifying any connected or IoT-enabled platform as an automated system.

Previous studies illustrate different levels of integration. Cho et al. [8] developed a system capable of automated calibration and on-site measurement of NO_3_^−^, K^+^, and Ca^2+^, although the system did not independently complete the entire nutrient dosing feedback loop. Pandey et al. [12] integrated multichannel sensing with PLC-based control logic and actuator operation, while Tatas et al. [27] demonstrated an IoT-based platform in which monitoring functions were linked with control actions. More advanced ion-specific approaches have moved closer to closed-loop nutrient management. Jung et al. and Cho et al. [11,29], for example, coupled estimated nutrient concentrations with fertilizer selection and dosing procedures, thereby linking sensing more directly with nutrient replenishment decisions. Cho et al. [64] also integrated an embedded controller, ion-selective electrodes, and a nutrient dosing algorithm for automated NO_3_^−^, K^+^, and Ca^2+^ management, with validation during lettuce cultivation.

Despite this progress, the literature remains uneven in terms of the extent to which the complete control chain is validated. Basic sensing, communication, and dosing functions are commonly demonstrated, whereas automatic recalibration, independent confirmation of actuator performance, structured recovery from faults, full crop cycle validation, and commercial-scale operation are reported much less consistently. Therefore, future evaluations should report not only whether sensing and control are integrated but also which functions are automated, independently verified, and capable of maintaining safe operation during faults or communication failures. Table 8 compares representative system architectures according to sensing, controller platform, actuation, control-loop completeness, validation scale, and failure handling provisions.

### Commercial Benchmarks and Economic Evaluation

Commercial equipment provides an engineering benchmark for prototypes. Priva describes NutriJet with up to ten fertilizer channels, dual EC/pH measurements, and irrigation capacities from approximately 5 to over 80 m^3^ h^−1^ [65]. Bluelab documents EC/pH dosing with configurable dosing and mixing intervals, alarms, safety lockouts, and restart after power loss [32]. These manufacturer-described functions establish a comparison checklist; they do not provide independent evidence of ion-specific accuracy, uptime, or cost savings. The basil study by Bhat et al. [10] includes a commercial dosing treatment, but its headline yield and water use improvements are relative to manual fertigation. Superiority over the commercial comparator should therefore be established from the relevant treatment contrasts, not inferred from those percentages.

Economic evaluation should report installed sensor, controller, dosing, plumbing, and commissioning costs in a stated currency and price year. Annual operating costs should include standards, reagents, cleaning supplies, replacement parts, calibration and maintenance labor, power, connectivity, and downtime. Compare alternatives at the same production scale, crop, nutrient targets, and service period, reporting cost per cubic meter of solution managed and per kilogram of marketable crop. Payback calculations require measured fertilizer, water, labor, and yield differences against a stated baseline, with sensitivity to labor price and sensor replacement interval. Comparable cost and service life data are insufficient in the present evidence synthesis; economic superiority cannot be assigned from component price alone.

## 9. Limitations and Future Directions

The limitations identified throughout this review indicate that future research should move beyond incremental improvements in individual sensors or control algorithms toward the systematic development of reliable, validated, and deployable nutrient management systems. Rather than reiterating previously discussed issues such as sensor drift, fouling, matrix interference, hydraulic uncertainty, and fertilizer–ion coupling, these challenges can be organized into a prioritized research roadmap. Near-term priorities should establish trustworthy continuous sensing and actuation; medium-term priorities should demonstrate robust multi-ion performance under realistic and standardized greenhouse conditions; and long-term priorities should integrate predictive control with commercial-scale validation.

Table 9 proposes measurable evidence milestones for future studies. The horizons indicate research sequence rather than promised calendar deadlines. Acceptance tolerances must be specified before testing for each crop, device, and operating range; the milestones are proposed validation requirements, not achievements established by this review.

### 9.1. Near-Term Priorities: Reliable Sensing and Actuation

Near-term research should improve measurement reliability through automated calibration and sensor health diagnostics. Hydroponic systems have already demonstrated automated sampling, rinsing, calibration, and normalization, but drift, reference instability, and loss of sensitivity remain important concerns [8,9,14]. Future systems should therefore be able to identify unreliable measurements before they are used for control. A specific target is a solid contact ISE array with a stable reference, suppressed water layer formation, and automated checks that extend the validated calibration interval. Claims of calibration-free operation should require independent reference measurements through a full crop cycle, rather than extrapolation from a short calibration test [23,31].

Actuation should also be independently verified. Flow or delivered volume measurements, stock tank status, valve state confirmation, and dose limits can help detect pump or injector failures that would otherwise convert a correct control command into an incorrect nutrient addition [53,54].

### 9.2. Medium-Term Priorities: Multi-Ion Robustness and Standardized Validation

Medium-term research should focus on multi-ion calibration that remains valid across changing nutrient formulations, crop stages, source water compositions, and ionic conditions. Sensor array and multivariate approaches are promising, but their performance must remain stable as the nutrient matrix and sensing elements change over time [14,24,66]. Benchmark datasets should span crop stages, source water mixtures, fertilizer formulations, and interfering ion ratios, with validation separated by cultivation run or site to prevent calibration leakage. The priority is robust transfer of calibration rather than a higher within-dataset R^2^ alone.

Standardized greenhouse validation is also needed because current studies differ widely in calibration procedures, experimental duration, reference methods, and reported metrics. Common reporting of analytical error, drift, calibration frequency, uptime, maintenance, and cultivation duration would allow for more meaningful comparison among technologies [9,17,42].

### 9.3. Long-Term Priorities: Predictive Control and Commercial Validation

Long-term development should move from reactive nutrient correction to predictive and coordinated control. Model-based approaches could combine current ion concentrations with plant uptake, water use, fertilizer composition, solution volume, and operational constraints to determine suitable dosing actions [11,67]. A control transfer framework should separate crop nutrient targets and uptake models from equipment-specific pump gains, mixing delays, and sensor calibrations. Transfer to a new crop or hydroponic configuration should follow bounded re-identification and independent validation under dilution, sensor bias, and actuator failure tests, with explicit criteria for when retuning is required.

Finally, technologies should be validated over complete crop cycles and across different greenhouse facilities, crops, seasons, and nutrient management conditions. Such studies should report maintenance requirements, component failures, downtime, operating cost, and fault recovery to establish whether a system is suitable for routine commercial deployment [19].

### 9.4. Technology Readiness and Deployment Evidence

EC, pH, temperature, level, and basic flow sensing, together with conventional PLC- or embedded-controller-based dosing, represent the most mature technologies for routine greenhouse application. In contrast, ion-selective arrays, spectroscopic nutrient estimation, sensor fusion platforms, and individual ion dosing remain supported mainly by laboratory, prototype, pilot, or limited greenhouse validation. Long-duration, multisite evidence is therefore still needed before unattended commercial maturity can be established. The reviewed studies also differed substantially in crop species, hydroponic configuration, nutrient formulation, calibration procedure, validation duration, reference method, controller metric, and greenhouse scale. Consequently, direct numerical comparisons should be interpreted as a comparative evidence synthesis rather than standardized technology certification. Table 2 is intended to make this interpretation auditable. Incomplete reporting of maintenance frequency, service life, or cultivation duration lowers confidence in a readiness assignment and must remain visible rather than being replaced by a presumed score.

### 9.5. Sustainability and Integrated Plant Health

Future nutrient control studies should address ecosystem-based resource management beyond adaptation to changing climate alone. Within the greenhouse boundary, this includes balancing nutrient supply with crop demand, accounting for solution reuse and discharge, and coordinating fertilization with beneficial microbial processes and pathogen management. Recirculation can conserve inputs while also requiring attention to biological risks; its environmental value must be evaluated together with crop health and the surrounding resource system [60].

Maintaining diverse cultivars and economically important plant genetic resources is a potential extension of controlled cultivation. A research pathway is to test whether genotype-specific nutrient control supports healthy propagation and ex situ maintenance of selected accessions. Such use would require separate evidence of genetic identity, reproductive or vegetative viability, and retention of diversity; nutrient automation alone does not establish preservation of a plant gene pool. Transfer to forestry or agroforestry should be investigated in relevant propagation or nursery systems using species-specific protocols, rather than assuming that greenhouse dosing algorithms apply directly to whole ecosystems.

## 10. Conclusions

The integrated framework supports an evidence-based deployment decision: validate the measurement, deliver a bounded dose, allow mixing, and independently verify the response. Each of the four evaluation levels is necessary; a sensor with strong laboratory regression or a connected dashboard does not by itself establish reliable crop cycle control.

Current evidence supports routine bulk EC/pH management and system-specific demonstrations of ion-resolved monitoring and dosing. Heterogeneous endpoints, short experiments, incomplete maintenance reporting, and limited search coverage prevent a universal accuracy estimate, a ranking of controller superiority, or a defensible commercial cost advantage. Results from related research platforms require independent replication before broad transfer is inferred.

The next decisive evidence is a full crop cycle comparison against a stated baseline with reference method checks, delivered dose measurements, fault tests, and recorded labor and consumables. Independent multisite studies should then test whether calibration and control transfer across cultivars and hydroponic configurations. Plant health and environmental outcomes should be assessed alongside control reliability; genetic resource maintenance remains a separate proposed application requiring validation.

## Figures and Tables

**Table 1:** Nutrient monitoring technologies used in hydroponic crop production.

Technology	Target	Principle	Advantages	Limitations	Greenhouse Readiness	Citation
EC	Total ionic strength	Conductivity cell	Fast, mature, inexpensive, continuous	No ion specificity; temperature sensitivity; deposits; air bubbles	High	[20,21]
pH	Hydrogen-ion activity	Glass or solid-state electrode	Direct control input; mature	Reference drift, fouling, frequent calibration	High	[20,21]
Temperature	Solution temperature	Thermistor or RTD	Stable and low cost; enables compensation	Location and response lag	High	[21]
Dissolved oxygen (DO)	Oxygen concentration	Optical or electrochemical probe	Direct root zone aeration indicator	Sensing cap or membrane maintenance and fouling	High for DWC; moderate for other hydroponic configurations	[21,22]
ISE/solid contact ISE	Individual ions	Potentiometric membrane	Ion-specific and potentially continuous	Cross-sensitivity, drift, reference stability	Moderate; technology-dependent	[8,23]
UV–Vis/colorimetry	Nitrate or reagent-specific analytes	Absorption/color reaction	Potential specificity and multiplexing	Matrix effects, optical fouling, reagents	Low to moderate	[24,25]
Multi-sensor	Multiple ions and system states	Sensor array plus multivariate model	Uses complementary responses	Calibration transfer and model drift	Prototype to pilot	[26]
IoT monitoring platform	Integrated sensor streams	MCU/PLC, network, and database	Remote records, alarms, and integration	Network, security, and data quality dependence	Moderate	[27]

**Table 2:** Evidence gates for interpreting greenhouse readiness in this review.

Descriptor	Validation Evidence Required	Maintenance and Integration Evidence
Prototype to pilot	Laboratory proof of concept or short integrated demonstration; no sustained cultivation evidence.	Calibration or model performance shown; routine maintenance and recovery remain unverified.
Low to moderate	Representative nutrient matrices and at least limited pilot or cultivation validation.	Interference/drift considered; sustained maintenance interval or autonomous recovery unresolved.
Moderate	Repeated operation during cultivation in an actual greenhouse or indoor hydroponic system; duration explicitly reported.	Calibration/cleaning procedures documented; sensor data linked to the intended monitoring or control task.
Moderate to high	Full crop cycle use with representative environmental and hydraulic variation.	Maintenance burden, data availability, and feedback/fault handling quantified; transfer remains to be established.
High	Routine greenhouse application supported by established practice and repeated crop cycle evidence for the device/application.	Documented calibration and consumables, operational reliability, and safe integration; no critical unresolved deployment gate.

**Table 3:** Operational evidence and unresolved maintenance information for continuous nutrient sensing.

Sensor Family	Deployment Evidence	Calibration and Maintenance	Lifetime and Cost Evidence
EC/pH	Routine control input, sensor interaction caused loss of reliable automated readings in a microscale system [30]	Standards/buffers, cleaning, junction inspection, and post-dose stabilization; intervals depend on drift and matrix	Comparable lifetime and annual maintenance cost not established across the cited systems
Conventional ISE	Real greenhouse paprika measurements with laboratory reference comparison [8]	Automated rinsing, calibration, and drift normalization; membrane and reference must be assessed independently	Study duration is not electrode service life; replacement intervals and cost need deployment logs
Solid contact ISE	Two-week indoor/greenhouse demonstration [23], screen-printed nutrient-solution application [31]	Multi-ion calibration and reference checks; solid contact does not eliminate membrane aging or fouling	Two-week operation does not establish crop cycle lifetime or calibration-free operation
Optical/spectroscopic	Microscale cultivation deployment [30], reagent-free NPK estimation [25]	Optical path cleaning, waterproofing, blank/reference checks, and model transfer; colorimetry adds reagents	Housing leakage was observed shortly after the spectroscopic experiment [30]; transferable replacement costs not established
Temperature/DO	Complementary root zone measurements in hydroponic monitoring [21,22]	Temperature reference checks; DO cap/membrane and fouling maintenance depend on probe type	Comparable sensor lifetime and maintenance expenditure not established by these crop studies

**Table 4:** Ion-specific nutrient sensing technologies for hydroponic solutions.

Sensor Family	Target	Principle	Application/Range	Interference/Limitations	Calibration/Deployment	Validation/Duration	Source
Conventional ISE	NO_3_^−^, K^+^, Ca^2+^	Membrane potential vs. reference electrode	Direct ion measurement; cited hydroponic validation covered approximately 40–1200 mg L^−1^	Competing ions, ionic strength, pH, temperature	Automated calibration demonstrated; moderate readiness for hydroponic monitoring	On-site hydroponic samples; comparison with ion chromatography	[8]
Solid contact ISE	NO_3_^−^, K^+^, NH_4_^+^, and other ions	Ion-selective membrane with solid ion-to-electron transducer	Application-dependent; real-time element-specific hydroponic monitoring demonstrated	Potential drift, water layer formation, membrane aging, interfering ions	Array-compatible and IoT-ready; long-term robustness remains a constraint	Two-week indoor and greenhouse cultivation	[23,31]
ISFET ion-sensitive FET	Primarily pH; functionalization can extend ion targets	Ion-dependent surface potential modulates transistor current	PO_4_^3−^, NO_3_^−^, and K^+^ demonstrated in nutrient solutions	Reference stability, drift, fouling, cross-sensitivity	High miniaturization potential; prototype/specialized use	Laboratory nutrient solution validation	[33]
Reagent/electrochemical sensor	Nitrate, phosphate, or other analytes	Potentiometric, amperometric, or voltammetric reaction	Method-dependent; phosphate measurement demonstrated in hydroponic solutions	Matrix effects, interfering ions, reagent/ electrode condition, aging	Controlled sampling, calibration, and electrode maintenance; low to moderate readiness	Laboratory or controlled sampling hydroponic validation	[28,29]

**Table 5:** Proposed four-level framework for evaluating monitoring technologies for continuous greenhouse monitoring and control.

Evaluation Level	Indicators	Technical Case and Evidence Boundary
Analytical	bias, RMSE or other prediction error, sensitivity, selectivity, limit of detection, repeatability, measurement uncertainty, agreement with a reference method	Analytical: Reference [8] compared ISE outputs with ion chromatography; high R^2^ coexisted with slopes departing from unity. Report n and ion-specific ranges.
Temporal	response time, recovery time, stabilization time, drift rate, calibration interval	Temporal: Reference [23] monitored nutrients for two weeks. Exposure duration alone does not establish drift rate, stabilization time, or electrode life.
Operational	fouling resistance, cleaning requirement and interval, maintenance frequency and labor, consumable demand, sensor lifetime, robustness to temperature variation, hydraulic fluctuations, bubbles, and solution matrix changes	Operational: Reference [30] required manual EC/pH measurements after sensor interference. Record maintenance, downtime, and failure recovery.
System/control	Missing data rate, uptime, communication latency, synchronization, invalid data rejection, fault detection, actuator response, settling time, overshoot, control stability	System/control: Reference [11] evaluated five replenishment steps; Reference [27] observed transmission failures during approximately 1000 h. Neither establishes commercial nutrient control uptime.

**Table 6:** Integrated comparison of nutrient control strategies, reported quantitative evidence, and required benchmarks.

Strategy and Scenario	Response and Disturbance Rejection	Reported Error	Tuning Burden and Limitation	Comparison Metrics and Sources
Threshold/on–off: EC/pH bands	Limited by dosing, mixing, and delay	EC tolerance ≈ 0.05 mS cm^−1^ [43]	Low–moderate *; overshoot and cycling	Settling time, overshoot, time within band, recovery [13,43,44]
PI/PID: setpoint tracking	Integral action compensates sustained disturbances	No comparable error benchmark extracted	Moderate–high *; system-specific tuning	Settling time, steady-state error, overshoot, recovery [13,45]
Rule/fuzzy/ANFIS: nonlinear control	Depends on rules and training conditions	pH: 1–3 steps; TDS: 1 step; 60 s/step [46]	Moderate–high *; limited transferability	Response time, residual error, overshoot, recovery [46,47,48]
Ion-specific dosing: composition correction	Repeated dosing, mixing, and measurement	Single test: NO_3_^−^ 8.0 ± 11.0%; K^+^ 7.9 ± 2.1%; Ca^2+^ 10.6 ± 8.0% [11]; ratio error: 5.9% [49]	High *; calibration and coupled fertilizer chemistry	Per-ion error, dosing error, iterations, mixing time [11,29,49]
Model-based/MPC: constrained dosing	Depends on model and state estimates	No comparable experimental error benchmark extracted	High *; model identification and computation	Tracking error, constraint violations, computation time, resource use [50,51]

* Review judgment.

**Table 7:** Representative quantitative performance of hydroponic nutrient control approaches.

Approach	Controlled Variable	Reported Performance	Validation Context	Interpretation/Limitation	Citation
Transport/mixing-aware EC control	EC	Control within approximately 0.05 mS cm^−1^	Multichannel greenhouse nutrient mixing system	Demonstrates importance of transport and mixing; not an ion-specific accuracy metric	[43]
Stepwise ion-specific replenishment	NO_3_^−^, K^+^, Ca^2+^; solution volume	8.0 ± 11.0%, 7.9 ± 2.1%, 10.6 ± 8.0%, 4.2 ± 3.7%, respectively	Representative stepwise replenishment experiment	Single study; over/under-injection in some steps; not evidence of long-term commercial reliability	[11]
Hybrid EC + ion-equivalent ratio control	Ionic composition target ratio	Final target ratio error 5.9%; reported 58% improvement over EC-only replenishment	Closed hydroponic nutrient management study	Supports hybrid bulk + ion-specific feedback; performance remains system- and formulation-dependent	[49]
Mamdani fuzzy NFT control	pH and TDS	pH: 1–3 control steps; 60 s per step; TDS: one control step	NFT experimental control study	Step duration is not a universal settling time; no pooled ion-specific error or commercial reliability estimate	[46]
IoT fertigation with a commercial comparator	Basil yield and water use efficiency	Compared with manual fertigation: yield +19%; water use efficiency +18%.	2022–2023 basil study; manual, commercial doser, and developed IoT doser treatments	Cultivation outcomes are not controller tracking errors; the reported percentages compare with manual fertigation, not the commercial doser	[10]

**Table 8:** Representative integrated nutrient monitoring control architectures for hydroponic systems.

Architecture	Sensing	Controller/Platform	Actuation	Control-Loop Status	Validation	Feedback and Failure Handling	References
EC–pH dosing control	EC, pH, solution level	Embedded controller/PLC	Water, nutrient, and acid/base pumps	Closed-loop bulk control	Experimental greenhouse systems	EC/pH measurement, limited actuator verification and failure handling	[43,44]
IoT monitoring and control	EC, pH, level, operational sensors	MCU/PLC and IoT platform	Dosing and circulation devices	Remote monitoring and automated control	Prototype/pilot systems	Depends on calibration, connectivity, interlocks, and recovery	[27]
Ion-specific monitoring	NO_3_^−^, K^+^, and Ca^2+^ ISEs	Automated calibration and acquisition	None	Monitoring only	On-site hydroponic validation	Verified using ion chromatography, no actuator verification	[8]
Ion-specific dosing	NO_3_^−^, K^+^, Ca^2+^, and volume	Dosing/fertilizer selection algorithm	Multiple dosing pumps	Prototype closed-loop control	Limited greenhouse validation	measurement feedback; affected by dosing error, ion coupling	[11,29]

**Table 9:** Prioritized research roadmap for nutrient monitoring and control technologies.

Priority	Research Requirement	Proposed Measurable Evidence
Near-term	Automated calibration and stable reference ISE arrays	Validate error and drift over one crop cycle; extend calibration intervals at the same predefined error tolerance.
Near-term	Dose verification and hard dosing limits	Measure each delivered dose; test pump, flow, and sensor faults; confirm zero dosing commands after lockout.
Medium-term	Multi-ion calibration transfer	Validate on an excluded run or site; report per-ion error, bias, and recalibration without validation data leakage.
Medium-term	Standardized greenhouse validation	Document test conditions and raw data; report settling time, overshoot, missing data, and maintenance labor.
Long-term	Predictive dosing and transfer across systems	Compare against a matched baseline; report tracking error, constraint violations, resource use, computation, and retuning.
Long-term	Commercial validation	Test one crop cycle at two independent sites; report uptime, calibration, replacements, recovery, and annual cost with uncertainty.

## Data Availability

No new data were created or analyzed in this study. Data sharing is not applicable to this article. All data are contained within the article.
